# Pharmacological Treatment for Social Cognition: Current Evidence

**DOI:** 10.3390/ijms22147457

**Published:** 2021-07-12

**Authors:** Cecilia Riccardi, Cristiana Montemagni, Elisa Del Favero, Silvio Bellino, Claudio Brasso, Paola Rocca

**Affiliations:** Department of Neuroscience “Rita Levi Montalcini”, University of Turin, 10100 Turin, Italy; cecilia.riccardi@unito.it (C.R.); cristiana.montemagni@unito.it (C.M.); elisa.delfavero@unito.it (E.D.F.); silvio.bellino@unito.it (S.B.); claudio.brasso@unito.it (C.B.)

**Keywords:** social cognition, schizophrenia, pharmacological treatment, antipsychotic

## Abstract

Cognitive impairment is currently considered a core feature of schizophrenia (SZ) and is gaining attention as a fundamental therapeutic target. Standard treatment for SZ involves the use of antipsychotics that are successfully used to control positive symptoms and disorganized behaviour. However, it is still unclear whether they are effective on social cognition (SC) impairment. Furthermore, different medications are currently being studied to improve SC in patients with SZ. A literature search on this topic was conducted using the PubMed database. All kinds of publications (i.e., reviews, original contributions and case reports) written in English and published in the last 15 years were included. The aim of our literature review is to draw a picture of the current state of the pharmacological treatment of SC impairment in SZ.

## 1. Introduction

Schizophrenia (SZ) is a major psychiatric disorder. It is characterized by the disruption of various mental processes, including the perception of reality, emotions and cognition. Standard treatment involves the use of antipsychotics that are successfully used to control positive symptoms and disorganized behaviour. However, cognitive impairment, especially social cognition (SC), is gaining attention and is considered by some psychiatrists as the core feature of SZ; for these clinicians, future treatments should target SC deficits as important limits for recovery from SZ.

Historically, it can be argued that cognition has been a focus for researchers since Bleuler, Kraepelin and Jaspers over 100 years ago [1,2,3]; however, it started to be the target of pharmacological and psychosocial treatment in more recent years. Over the past two decades, research on cognition in SZ has widely expanded, highlighting new important information about this illness. First, between 75 and 85% of patients with SZ show low cognitive performance and, interestingly, their family members also exhibit mild cognitive impairment [4,5]. Second, Reichenberg and colleagues [6] pointed out that the impairment of certain cognitive domains is present in the prodromal phase of SZ before the onset of psychotic symptoms and it progressively worsens over the course of the illness. Furthermore, according to Green and colleagues [7], the degree of cognitive impairment directly reflects the future functional outcome of patients with SZ.

Cognition has two branches: neurocognition (NC) and SC. NC includes mental abilities such as working memory, learning and memory, attention, processing speed, reasoning and problem solving [8]. For a long time, NC has been considered the main predictor of functional outcomes [9,10,11]. However, the other branch, SC, is gaining considerable attention. It refers broadly to the high mental processes involved with perception, storage and the use of social information that helps everyone to make sense of themselves and others. Increasing evidence highlights that SC is also a direct predictor of functional outcomes, particularly of community and social functioning such as to fulfil basic social roles and being involved in social relationships [12]. NC and SC are interlinked. Indeed, SC may act as a mediator between basic neurocognition and daily life functioning [13,14]. Several studies, by using path analysis and structural equation modelling, have highlighted that the explanation of functional outcome variance of patients with SZ depends more on SC than on NC [13,15,16,17,18,19]. Moreover, social neuroscience studies of SZ have shown functional and structural abnormalities in brain areas associated with specific social cognitive domains [20]. These findings directed researchers toward specific treatments for the impairment of SC in order to obtain functional changes in the daily life of patients with SZ [21].

Numerous psychosocial interventional approaches have been developed with the aim of improving aspects of SC [22,23]. At first, training for SC was embedded among treatment for neurocognition and this resulted in difficulties in understanding whether the improvement on SC was specifically due to social cognitive tasks or to the global approach. Broad-based interventions included SC training within broad psychosocial approaches [24,25,26].

For example, social skills training, which is one of the most employed training for SC, does not consist of specific practices for implementing underutilized social cognitive processes as it helps individuals to acquire and practice specific behavioural skills in social interactions. A few treatment packages have included SC training exercises as one element of much broader training programs: Integrated Psychological Therapy [27] and Cognitive Enhancement Therapy [28] are multi-element treatment packages that includes extensive SC training along with cognitive remediation and social skills training. They both revealed moderate effects on SC outcomes [29]. The first one affirms that basic NC deficits have a pervasive effect on higher levels of behavioural organization, including social skills and social functioning [30], while the second one is based on the hypothesis that NC deficits in several cognitive clusters caused impairment on encoding, remembering, interpreting and responding to subtle cues regarding context-specific rules or affect [31,32].

Recent broad-based interventions incorporate novel techniques (computerized training, online training programs and virtual reality), are shorter in duration and do not target specific social cognitive domains [26]. This type of social cognitive training focused on the extended practice of elementary social cognitive skills programs and has provided satisfactory results with respect to both SC and social functioning.

Later on, researchers concentrated on specific social cognitive domains, demonstrating that it was possible to improve performance on social cognitive tasks. A series of targeted interventions are focused on delivery of SC treatment without any other intervention components. An example is the Cognitive Remediation (CR), which boosts information processing skills as a means of indirectly improving social functioning. CR is defined as “a behavioural training-based intervention that aims to improve cognitive processes (attention, memory, executive function), social cognition and metacognition, with the goal of durability and generalization” (Cognitive Remediation Experts Workshop (Florence, Italy, April 2010)) [33]. It is based on targeted training strategy that could be personalized. CR and targeted interventions, in general, have reported positive outcomes in improving SC, particularly in the domains of emotion processing and theory of mind, but their effects on social functioning have not been universally confirmed [26]. Recently, comprehensive training with tasks of multiple social cognitive domains have been designed and obtained promising results [34].

It remains an open question whether the psychopharmacological approach is a viable solution to treat cognitive impairment. Due to the complexity of understanding the biological bases of SC, the development of pharmacological intervention is still limited. Therefore, both basic and clinical research are now focusing on this topic. The aim of our literature review is to draw a picture of the current state of pharmacological treatment for SC impairment in SZ. In order to do so, we first presented a quick overview on what SC is, how it is assessed, its impairment and its neural correlates. Then, we focused on exploring the current ideas on pharmacological treatment of social cognitive impairment in SZ.

## 2. Overview of Social Cognition in Schizophrenia

### 2.1. Social Cognition

SC refers to psychological processes involved in the perception of social signals and in the connection of such perception to motivation, emotion and adaptive behaviour. It allows people to recognize, manipulate and behave with respect to socially relevant information. According to Adolphs [35] “Social cognition guides both automatic and volitional behaviour by participating in a variety of processes that modulate behavioural response: memory, decision-making, attention, motivation and emotion are all prominently recruited when socially relevant stimuli elicit behaviour.”

At the National Institute of Mental Health workshop in 2006 [36] it was stated that SC includes five principal domains: Theory of Mind, emotional processing, social knowledge, social perception and attribution bias.

1. Theory of Mind (ToM): ToM is the ability to attribute mental states, such as desires, intentions and beliefs, to others and to understand that others have beliefs that are different from one’s own [21]. Skills that help to understand the intentions of others, such as non-verbal communications and sarcasm, are included in ToM [37]. Deficits in ToM may cause misreading of intentions, emotions or cues from others, with consequent difficulties in social communication and limited expression of empathy toward others. Later, the study of ToM has been extended to SZ patients based on evidence that alterations in SC may play a key role in the pattern of their clinical symptoms [38]. In the early 1990s, studies conducted by Frith and colleagues [39,40] and by Corcoran [41] supposed that the deficit of people with SZ in the understanding of oneself and the mental states of others underlying overt behaviour (i.e., ToM or mentalization deficit) resulted in the inability in creating metarepresentations that correctly attribute thoughts to others. According to these authors, this difficulty would represent a core feature in the genesis of psychotic symptoms, therefore ascribing mentalization processes a key role in the psychopathology of SZ [42,43].
2. Emotional processing: Emotional processing is the ability to perceive and use emotions. It defines the emotional intelligence of an individual and includes the capacity to identify, understand and manage emotions [44].
3. Social knowledge: Social knowledge is the awareness of the rules that define interpersonal relationships in society [45]. It is fundamental for people to reach adequate social competence [46], defined as the capacity to solve interpersonal problems through verbal and non-verbal communication [47].
4. Social perception: Social perception is the ability to identify, understand and employ social cues, roles and rules to make inferences about context, relationship and social situations using verbal and non-verbal communication [48]. It allows people to form impressions of others from elements of social interaction [49].
5. Attribution bias: According to Kelley [50], human behaviour can be usually attributed to internal factors, such as one’s will and intentions; or to external factors, such as situations. Using causal attribution human beings judge or infer reasons for the behaviour or social situations of others. An attribution bias is an error in attributing a cause to internal or external factors.

### 2.2. Assessment of Social Cognition in SZ

In 2003, the National Institute of Mental Health (NIMH) established the Measurement and Treatment Research to Improve Cognition in Schizophrenia (MATRICS) initiative to clarify the main concepts and evaluation criteria associated with NC and SC relative to people living with SZ. The goal of the MATRICS consensus conference (MCC) was two-fold: First, to identify cognitive domains worthy of attention in a consensus cognitive battery; second, to establish criteria for selections of the battery tests [51]. SC was included as one of the seven domains represented in the MCC Battery (MCCB) for clinical trials in SZ and the Managing Emotions component of the Mayer–Salovey–Caruso Emotional Intelligence Test (MSCEIT) was selected [36,52].

There are other tests used to assess the specific domain of SC, such as difficulty in recognizing facial emotion. For example, Facial Emotion Discrimination test, Facial Emotion Identification Test (FEIT) [53] and Penn Emotion Recognition Test (ER-40) [54]. The ER-40 is considered a promising tool to evaluate emotion identification and response in SZ [55]. In addition, the Bell Lysaker Emotion Recognition Task (BLERT) evaluates emotion processing through the recognition of seven emotional states [56].

The Awareness of Social Inference Test (TASIT) is a ToM test designed with seven scales: positive emotions, negative emotions, sincere, simple sarcasm, paradoxical sarcasm, sarcasm enriched and lie. The scales are organized into three sections: emotion recognition, social inference minimal and enriched [57].

Mental state attribution can also be investigated through the Reading the Mind in the Eyes Test (Eyes) and the Hinting Task. Eyes focuses on the capacity of understanding the mental states of others from expressions in the eye region of the face [58]. Hinting Task focuses on the ability to infer the true intent of indirect speech [41].

The Social Cognition Screening Questionnaire (SCSQ) is designed to assess multiple domains of SC, especially ToM, metacognition and hostility bias. The SCSQ presents interpersonal vignettes that describe ambiguous interpersonal situations and the subject is requested to answer yes/no questions. Moreover, the SCSQ can assess non-SC as schematic inference and verbal memory [59,60].

The Intentional Bias Task (IBT) evaluates the tendency to attribute intentionality to the actions of others. Patients are asked to define action as occurred “on purpose” or “by accident” [61].

The Mini Profile of Nonverbal Sensitivity (MiniPONS) and The Social Attribution Task-Multiple Choice version (SAT-MC) are used to assess social perception by decoding interpersonal cues [62,63].

Even though these tools are currently used to evaluate SC in SZ, it can be argued that it is still missing a strong validation of measures to assess SC and this represents a significant limitation for clinical trials [64]. The Social Cognition Psychometric Evaluation (SCOPE) study was designed to reduce this limitation [64,65] by systematically evaluating the psychometric properties of promising measures. It was a five-phase project that ultimately focused on the following tasks: BLERT, ER-40, Eyes, TASIT, Hinting Task, MiniPONS, SAT-MC and IBT. According to its results, the BLERT, Hinting Task and ER-40 are recommended for use in trials as they possessed the strongest psychometric properties; Eyes, IBT and TASIT require further study as they possessed weaker psychometric properties; and MiniPOS and SAT-MC have poorer psychometric properties.

### 2.3. Social Cognition Deficit in Schizophrenia

SZ patients show meaningful deficits in different dimensions of SC [66]. These deficits are considered core features of SZ and seem to be present throughout the course of the disease from the prodromal phase in high-risk subjects, in the first stages of the illness and during symptom remission. They were also demonstrated in first-degree relatives of SZ patients [13,67,68,69,70]. These findings suggest that SC deficits are unlikely to be an undesirable effect of taking psychoactive medication [71].

Recently, a comprehensive review of SC in first episode psychosis (FEP) examined 48 relevant studies and showed consistent SC deficits in individuals who experienced a FEP, particularly in emotional processing and ToM [72]. Furthermore, SC deficits appear to be stable over time in FEP samples and comparable with SZ clinical groups.

According to Galderisi and colleagues [73] and previous studies [14,74], the impairment of SC in patients with SZ could be a mediator of the relationship between NC and functioning. Furthermore, according to the meta-analysis of Fett and colleagues [13], SC, as compared to NC, could have a stronger connection to functional outcome.

Recently, Rocca and colleagues [75] conducted a study that stratified the functional outcomes of patients with SZ on the basis of their impairment in SC. They used a large patient sample recruited in the context of the Italian Network for Research on Psychoses (NIRP). A total of 809 patients with SZ completed the SC assessments that included MSCEIT, TASIT and FEIT. It also evaluated NC, psychopathology, real life functioning and the milestones reached. With cross-sectional data, they identified three clusters based on SC: unimpaired (42%), impaired (50.4%) and very impaired (7.5%). Their findings showed that real life functioning worsened significantly from the unimpaired to the impaired and very impaired cluster, denoting a strong correlation between SC and functioning.

### 2.4. Neuroanatomical Substrates of Social Cognition in Healty Subjects and in Peolple with SZ

About thirty years ago, Brothers was the first to define the existence of a “social brain”. He sustained that the network by which social knowledge operates is different from that of other types of knowledge [76]. Studying primates, he suggested that the “social brain” consists of three regions: the amygdala, the orbitofrontal cortex and the temporal cortex. Thanks to functional neuroimaging, particularly functional magnetic resonance imaging (fMRI), more brain areas were added to the “social brain”: the medial prefrontal cortex (MPFC), the inferior frontal gyrus (IFG), the interparietal sulcus, the inferior parietal lobule (IPL), the anterior insula (AI), the anterior cingulate cortex (ACC), the posterior cingulate cortex/precuneus (PCC/PC) and the amygdala (Amy) [38,77,78,79]. Furthermore, specific areas of the temporal cortex were found to be involved in fMRI SC tasks: the temporoparietal junction (TPJ), the posterior superior temporal sulcus (p-STS) and the fusiform gyrus (FFG). In addition to these areas, the mirror neuron system (MNS) is considered part of the “social brain” [80] and is embedded in the IFG, in the ventral and dorsal premotor areas, in the supplementary motor area, in the STS, in the primary motor cortex, in the primary somatosensory cortex, in the posterior middle temporal gyrus (p-MTG), in the fusiform face area (FFA), in the IPL, in the middle temporal area (MTG) and in the AI [78,81,82,83].

Individually, these areas play a role that cannot be considered as purely social; however, together they shape the complexity of our social interactions. Among a wide range of skills, the “social brain” is responsible for the ability to assign emotional value to faces, to interpret expressions as fear and distrust, to process empathy-related stimuli and to understand the point of view of others in complicated social situations. In particular, some socio-cognitive tasks used in functional magnetic resonance imaging (fMRI) activate mostly cerebral areas involved in neuro-cognitive processes, while other tasks are associated with a higher involvement of brain area activated by affective stimuli and still others exhibit an intermediate pattern of activations [84]. In any case, a strong activation of language-related motor areas containing mirror neurons was found in all kinds of tasks suggesting that motor/mirror processes take place in most experimental paradigms assessing SC [84]. Moreover, when focusing on different fMRI tasks designed to study different domains of SC, some specificity of activated brain areas was found: ToM tasks were associated with the activation of the TPJ, MPFC, PCC/PC and of the anterior temporal lobe (ATL); social perception tasks with OFC, FFG and Amy activation; and social action observation with mental imitation tasks with IPL and IFG activation. The posterior superior temporal sulcus (pSTS) was involved in all different tasks [80]. Focusing on brain networks activation during SC fMRI task, two main types of cross-network interactions were reported: A negative coupling (segregation) between the Default Mode Network and the Control Network (composed by Ventral Attention, Frontoparietal and Dorsal Attention Networks) and a positive coupling (integration) between these two networks [85] according to the type of SC fMRI task proposed.

Focusing on neural correlates of SC in SZ, many studies have been conducted on people with SZ engaged in SC tasks while performing fMRI. This imaging was compared with that of healthy controls (HC) performing the same SC task in order to assess differences attributable to the mental disorder. From these kind of studies, PFC, FFG, right Amy, visual processing areas, ACC, IPL and STS altered activations were found in emotion recognition, processing and attribution tasks [86,87,88,89]; left posterior TPJ and STS were hypoactivated during ToM tasks [88,90,91], while altered activation where found in both types of SC fMRI tasks in the bilateral AI, in the right TPJ and in the left Amy [88]. In general, during SC fMRI tasks, people living with SZ showed hyperactivation of brain areas not directly involved in SC and hypoactivation of brain regions belonging to the social brain. This altered activation could be interpreted as a possible functional neural correlate of SC deficits observed in SZ. To confirm this speculation, further meta-analytic studies are needed to integrate this information about brain activations with a deeper knowledge of the role of functional networks interactions in SZ patients during standardized SC fMRI tasks exploring all the domains of SC.

## 3. Materials and Methods

A comprehensive search on PubMed database for articles in English published until 30 November 2020 was conducted. Search on PubMed was selected due to greater ease of research and greater availability of sources. Filters or MESH restrictions were not used. The search terms included were the following: “schizophrenia”, “social cognition”, “treatment”, “pharmacological treatment” and “antipsychotics”. The search covered the last 15 years.

Reviews, original contributions and case reports were included. The articles included dealing with the variation of SC impairment in people living with a SZ, schizoaffective and schizophreniform disorder after a pharmacological treatment assumption. Patients with a short duration of illness (even drug naïve patients) and patients with a history of chronic illness were included. Studies included evaluated at least one SC domain variation through the use of validated scales. Treatment with antipsychotic drugs have to last at least 2 weeks at an effective dosage, with no limitation for the number of doses and duration applied for the other drugs.

Overlapping papers, papers written in languages other than English and works that included psychotic features associated with other diagnoses (i.e., schizotypal personality disorder, major depressive disorder and bipolar disorders with psychotic features, substance/medication-induced psychotic disorder or psychotic disorder due to another medical condition) were excluded. References of relevant articles from screened records were retrieved to deepen the scope of our topic.

## 4. Results

**Figure 1:**
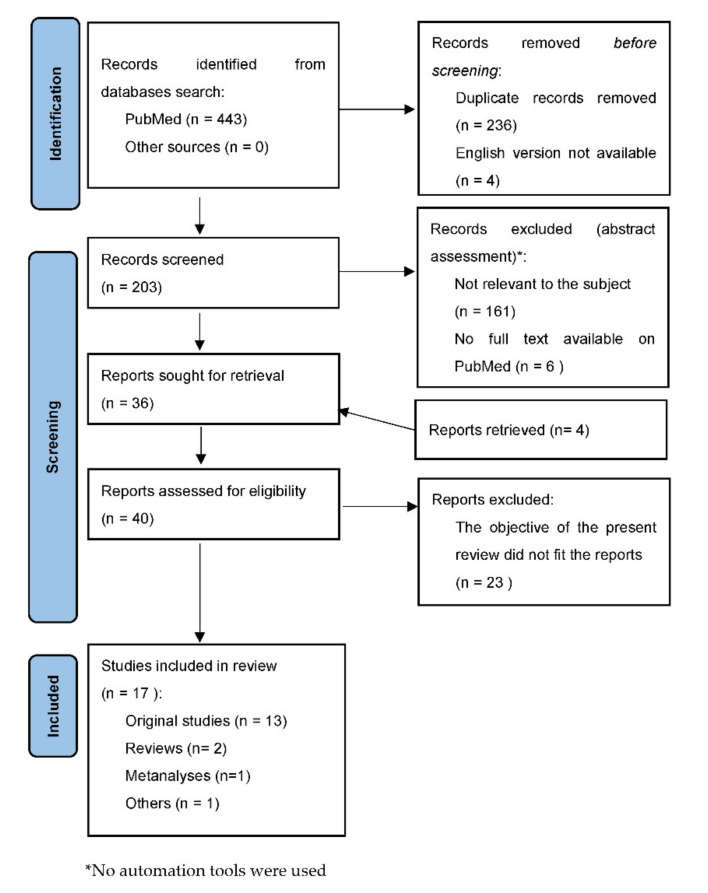
Flow chart of the search and selection of the literature reports.

Our search provided 443 results. By applying the criteria proposed, results were reduced at 203 articles. We retrieved other four interesting articles from the references of the screened records. As described in Figure 1, the final number of publications that were deemed eligible for the current review was 17. Among them 2 were narrative reviews, 1 was a meta-analysis, 1 was a leading article and 13 were original contributions (RCTs or observational studies). Table 1 shows the main characteristics and findings of the original contributions. No suitable case reports were found.

## 5. Discussion

Discussion is presented for every class of molecule exprerimented to improve SC in SZ.

### 5.1. Antipsychotics

Currently, antipsychotics are the main treatment for SZ. Several studies have specifically deepened the effects of these drugs (particularly, the second-generation antipsychotics) on SC impairment.

The mechanism of antipsychotics effects on SC is mostly unknown.

Second-generation antipsychotic drugs modulate dopamine neurotransmission and act as antagonists on serotonin 2A receptors (5-HT2AR). Inhibition of the 5-HT2AR serotonin receptors that are present on the cell bodies of dopaminergic neurons in the substantia nigra and ventral tegmentum may decrease dopamine release. Furthermore, projections of 5-HT2AR pyramidal neurons in the medial prefrontal cortex act as the modulator of mesocortico-limbic dopaminergic neurons [105]. In fact, they would follow the functional antagonism in the mesocortical pathway where the excess of dopamine causes positive symptoms, while the action as functional agonist in the mesocortical pathway improves the negative symptoms [106,107].

Different drugs have different affinities for target receptors determining the possible added desired or adverse effects of the different drugs: Multiple-acting receptor targeted antipsychotics (MARTAs) such as clozapine, olanzapine and quetiapine bind to multiple other neuroreceptors and have modest affinity to D2 and 5-HT2A; serotonin and dopamine antagonists (SDAs) such as risperidone, paliperidone, sertindole and lurasidone exhibit potent D2 and 5-HT2A antagonistic activities, with a high affinity for α1, 5-HT2C and H1 and negligible affinity for M1 receptors. Among them, risperidone presents the stronger dopaminergic D2 (with 72–81% striatal D2 receptor occupancy at a dose range of 4 to 12 mg/day) and serotonergic 5-HT2A antagonistic activities and show high affinity to adrenergic, 5-HT2C serotonin and H1 histamine receptors.

Finally, dopamine system stabilizers (DSSs) are partial D2, D3 and 5-HT1A-receptor agonists either as a functional agonist or a functional antagonist and antagonists at 5-HT2A receptors.

In addition to positive symptoms, serotonin and dopamine are thought to be involved in the improvement of SC and possible contributes to the improvement of emotional perception and social functioning, in particular, through the facilitating effect of serotonin antagonism on dopamine release in the prefrontal cortex [21,108]. Furthermore, the role of dopamine regulation in the mesocorticolimbic system mediated by second-generation antipsychotics could act as an emotional manager over the amygdala [109], this could explain the efficacy of SGAs compared to first generation antipsychotics on SC [110].

Maat and colleagues [111] conducted an 8 week, randomized, multicentre and open-label study on 80 SZ patient aged 16–50 years treated with aripiprazole (maximum dose 30 mg) or risperidone (maximum dose 6 mg). Patients of both groups of treatment obtained better scores in social cognitive and neurocognitive tests at week 8 than baseline. Aripiprazole appeared significantly superior than risperidone in reaction times for emotional working memory, a specific SC task proposed by the authors. This improvement correlated with social functioning. Mizrahi and colleagues [92] noted an improvement in ToM after 2 weeks of administration of clozapine, risperidone, olanzapine or loxapine in 71 patients with psychotic disorder (SZ, schizophreniform or schizoaffective disorder), that were initially drug free. Koshikawa and colleagues [93] conducted a 6 month pilot, open-label and randomized controlled study on 30 patients with SZ, comparing the effects of two long-acting injection drugs, paliperidone and risperidone, on social functioning as the primary outcome and on SC as one of the secondary outcomes. The assessment of SC was carried out with the Social Emotional Cognition Task (SECT). Their preliminary results showed a better improvement in social functioning in patients treated with paliperidone. Instead, there were no significant differences in terms of the SECT accuracy. Behere and colleagues [94] studied a sample of 25 antipsychotic-naïve patients with SZ and assessed their ability to recognize facial emotions typically impaired in SZ before and after a short-term exposure to risperidone (mean duration of treatment 38.2 ± 17.1 days); they were all treated uniformly at 4 mg/day. At the follow-up assessment, facial emotion recognition abilities were improved, especially for the emotion of disgust. Sumiyoshi and colleague [95] studied a sample of 20 SZ patient and reported that after 6 months of treatment with perospirone (mean dosage 18,3 mg/day), verbal SC that was evaluated by script tasks was improved. Roberts and colleagues [96] compared the effects of olanzapine (117 patients with a mean dose of 15.6 mg/day) and quetiapine (106 patients with a mean dose of 455.8 mg/day) on SC and social functioning. To assess SC, they used the Social Cue Recognition Task (SCRT) that requires participants to view short videos of four interpersonal vignettes and answer “true” or “false” to propositions about them. Cue subscales are the following: Low emotion vignette, concrete cues; Low emotion vignette, abstract cues; High emotion vignette, concrete cues; and High emotion vignette, abstract cues. This study found an improvement after 6 months of treatment in both groups; the improvement was modest but significant in 3 out of 4 SC cue subscales (low emotion-concrete, low emotion-abstract and high emotion-concrete). According to the Kucharska-Pietura and Mortimer review [97], a better improvement in SC should be expected with clozapine than with other second-generation antipsychotics.

Currently, evidence of the impact of antipsychotic drugs on SC is inconclusive. Mainly due to inconsistencies in study designs, methodologies, standardized drug dosages, numerosity of test samples and lack of long-term assessments [21,97].

### 5.2. Other Drugs

Raloxifene is a first-generation selective estrogen receptor modulator (SERM) that behaves as an agonist in the brain and bone and as an antagonist in all the other tissues [112].

The effectiveness of raloxifene treatment in man and women with SZ was first evaluated on neurocognition. A randomized, double-blind and placebo-controlled crossover trial was conducted on a sample of 98 patients with SZ or schizoaffective disorder; in addition to their usual antipsychotic medications, they received 120 mg/day of raloxifene. After 6 weeks of treatment, participants who have assumed raloxifene were improved in attention/processing speed and memory [113].

The same team have subsequently evaluated the effects of raloxifene treatment at the same dosage on SC skills [114]. Ji and colleagues conducted a randomized, double-blind and controlled crossover trial in order to determine if and how the adjunction of raloxifene (120 mg per day orally for 6 weeks) to standard treatment in patients with SZ would alter the abnormal neural activity during an angry facial emotion recognition fMRI task. According to their results, it appears that raloxifene significantly increased the activation in the right hippocampus and left inferior frontal gyrus compared with the placebo, suggesting that this treatment might change neural activity in brain regions known to be associated to facial emotion recognition. More studies are needed to confirm these preliminary findings.

Considering that abnormal activity of the GABA neurons of the prefrontal cortex might be involved in cognitive impairment, Kimoto and colleagues found that lower levels of GAD67 mRNA and protein have been consistently found in the dorsolateral prefrontal cortex of subjects with SZ. Quantification of GAD67 and Zif268 mRNA levels in dorsolateral pre-frontal cortex area in patients with schizophrenia and healthy comparison using polymerase chain reaction showed an altered expression of the transcriptional regulatory factor Zif268 in schizophrenia subjects that may provide a potential mechanistic basis for impaired GABA synthesis in the illness [98].

On this basis, Buchanan [115] investigated if GABA neuroactive drugs might improve NC and SC in SZ. Sixty-four participants with the diagnosis of SZ enrolled in a 4 week, placebo-controlled and parallel group double-blind study to evaluate if an adjunctive treatment with 3 mg BID or 8 mg BID of MK-0777 (a partial agonist of GABAα2/α3) to one second generation antipsychotic medication may improve cognitive impairments in SZ. The MCCB was used to assess neuropsychological test performance and also the emotion management of SC (through MSCEIT) was evaluated. No improvement was shown when MK-0777 was administered with antipsychotic treatment. The improvement of GABA signaling alone is probably not enough to produce an amelioration in SC [21].

### 5.3. Oxytocin

Despite mixed results, the most promising effort in pharmacological approaches to SC impairment seems to be the one focused on oxytocin.

Oxytocin is an evolutionarily conserved hypothalamic neuropeptide; it counts nine amino acids and it is very similar to vasopressin. It is synthesized in the paraventricular, supraoptic and accessory nuclei of the hypothalamus and it is released into systemic circulation [99].

Oxytocin receptors are typical class I G protein-coupled receptors. They need magnesium and cholesterol to reach a high-affinity state. The gene sequence of this receptor has been identified also in rats, mice, pigs, bovines, sheep and monkeys and it is expressed in several tissues other than the brain [116]. However, in the brain, few areas can be identified with higher receptor density, specifically the nucleus accumbens and prelimbic cortex of prairie voles, the lateral septum of montane voles and the posterior bed nucleus stria terminalis [117,118]. It is also interesting to note that the receptor distribution is different between males and females in montane voles [119].

Oxytocin was initially known for its role in parturition and lactation and recently it has gained attention for being involved in regulating the social behavior of all vertebrates [8,120]. Several studies have investigated the effects of oxytocin in the central nervous system by demonstrating its role in bonding and maternal care, aggression, fear, anxiety and interpersonal trust [121,122,123]. Growing evidence suggest that oxytocin has as important role as a modulator in cortical processing and enhances the salience of social information by disinhibiting cortical circuits [116].

Particularly, the ability to make and maintain social bonds is fundamental to assure reproductive success, longevity and health in social mammals, including humans. Close social bonds are a proxy to a solid social support system. However, how oxytocin is mediating these processes remains unclear [124]. Part of the explanation could be that oxytocin reduce the stress response; in humans, Heinrichs and colleagues [125] administered a standard psychological stress test (TSST) and showed that the presence of a friend together with the administration of intranasal oxytocin correlated with the lowest salivary cortisol level when compared to groups with no friend present or no oxytocin administration.

Impairment in social behavior is a core symptom of many psychiatric disorders. Extensive evidence from studies on animal models demonstrates that oxytocin plays a key role in social recognition and bonding. In the last decade, in human-based research, there has been increasing interest in using oxytocin to treat social behavior deficits in psychiatric illnesses such as autism spectrum disorder and SZ. To date, researchers have focused on establishing the functional effects of oxytocin measuring endogenous concentrations and observing the effects of exogenous administration [126]. Furthermore, a field of interest is the identification of polymorphisms and epigenetic modifications of the oxytocin receptor gene; there is evidence that both appear to be associated to a lower gene expression in the temporal cortex and cerebellar areas in patients with autism and SZ. There is also a reduced volume in frontal regions and temporal-limbic areas in women with SZ [127,128,129].

While it is true that initially researchers have focused on different disorders including depression, anxiety and borderline personality disorder, nowadays the focus is on autism and SZ as both the mental disorders are characterized by profound social dysfunction [130,131]. Some studies have found low levels of oxytocin concentration in blood or cerebrospinal fluid samples of SZ patients [132,133,134]. SC performance is related to peripheral oxytocin level and so it has been proposed as a possible biomarker in at-risk states for schizophrenia [135,136].

Hollander and colleagues [137] were the first to experiment intravenous doses of oxytocin in patients with autism. They described a reduction in repetitive behaviors and an improvement in learning with regard to affective speech [138].

In the case of SZ, the intranasal administration of oxytocin to improve SC and social functioning has yielded mixed results [12]. A number of studies have highlighted how single or repeated intranasal doses of oxytocin can ameliorate performance in task of social cognition.

Davis [100,139], Guastella [101], Shilling and Feifel [102] and Brambilla [140] conducted studies utilizing single administration with promising results. In particular, Davis and colleagues [100] conducted a randomized, double-blinded and placebo-controlled clinical trial on 27 male patients with SZ. Two groups were trained: One received 40 international units of oxytocin 30 min prior to the training session and the other one received a dose of placebo. Training sessions focused on three domains of SC: facial affect recognition, social perception and empathy. It was a 6 weeks and 12 session training. At the end of the training, the oxytocin group showed significantly greater improvements in empathic accuracy than the placebo group and this better performance was maintained one month after the last drug administration. Brambilla and colleagues [140] examined the effects of 4 months of treatment with intranasal oxytocin in 31 subjects with SZ with a randomized, double-blind and placebo-controlled trial. Abilities of SC were tested with a battery of tests including MSCEIT. According to their results, oxytocin improved the performance on MSCEIT compared to placebo. These findings suggest that patients with deficits in the ability to process social information may use oxytocin to improve the salience of social information. Consequently, the increased salience enhances the ability to learn higher-level socially cognitive skills.

In this review, Bartholomeusz and colleagues [136] summarize the behavioural effects of oxytocin in schizophrenia-spectrum disorders: It comprises six studies that analyze the effect of a single-dose intranasal oxytocin on complex SC process, including social perception and ToM and interpersonal perception; ten trials in which short-term treatment (2–8 weeks) with twice-daily intranasal oxytocin is compared to the placebo; and one study [103] in which a 6 week daily intranasal treatment is combined with social cognitive training in an early psychosis sample. This review confirms the potential efficacy of oxytocin treatment in the enhancement of SC abilities in SZ, on the other hand it highlights the inconsistency of the results found due to great differences in the sample characteristics, methodology and outcome measures used in the studies analyzed.

A recent meta-analysis [104] considered 12 studies where patients with SZ were randomized to intranasal oxytocin vs. placebo. They found that oxytocin had a significantly greater effect on high-level SC (i.e., mentalizing and theory of mind) compared to low-level SC (i.e., social cue perception), suggesting that this treatment might have a selective effect on high-level SC. This result appears important to investigate a more focused target in future studies of intranasal oxytocin.

Cacciotti-Saija and colleagues [103] failed to replicate these results in their double-blinded randomized controlled trial using a twice-daily chronic dose of oxytocin. In fact, they found no evidence of social cognitive improvement. An explanation of this failing might arise from preclinical studies that highlighted that the improvement in social behavior obtained after an acute administration of oxytocin may be lost with chronic administration [141].

However, future studies are needed to identify the most effective treatment regimen with oxytocin and the subtypes of patients might benefit the most from it.

## 6. Conclusions

SC has been recognized as a valid treatment target in SZ.

In terms of the pharmacological treatment of social cognitive impairment in patients with SZ, the literature suffers from wide inconsistencies in study design. Most samples are small, medications doses are not standardized and the control of clinical variables is often inadequate. Moreover, it is missing a wide and validated set of tests to evaluate in the standardized manner of all SC domains.

Antipsychotics have demonstrated unreliable effects on SC. The initial enthusiasm for new medications, such as oxytocin, raloxifene and GABA neuroactive drugs, born from early phase studies has faded in phase III studies. Oxytocin remains the most promising approach, but it requires further and deeper investigation.

Presently, the pharmacological enhancement of SC in SZ appears promising but is still in its embryonic stages. It is hopeful that large-scale longitudinal investigations will clarify the questions regarding the role of SC on severity of symptoms and real-life functioning in patients with SZ. In order to do so, wide studies with rigorous RCT methods are required.

## Figures and Tables

**Table 1:** Original contributions about studies that investigate pharmacological treatment in SZ in relation to SC impairment.

Study	Sample Charateristics	Study Design	Treatment, Dose, Duration	Time of Assessment	SC Measures	Main Findings about SC
[92]	48 patients Age 16-50 Mean age: 25.48 Schizophrenia Inpatients and outpatients All stage of illness *	Randomized, multicenter, open-label study	Risperidone 1-6 mg or aripiprazole 7,5-30 mg	Baseline and after 8 weeks of treatment	Facial affect recognition Emotional working memory Emotional learning task	Scores on social cognitive and neurocognitive tests improved with both treatments. There were few differences between the two antipsychotics on social cognitive test-scores
[93]	Cross-sectional study: 71 patients (mean age 33.02) Longitudinal study: 17 patients (mean age: 31) Age 15-65 Schizophrenia, schizophreniform and schizo-affective disorder Inpatients and outpatients All stage of illness *	Cross-sectional study and longitudinal study	Typical and Atypical antipsychotics 6 weeks	Baseline and after 2-4-6 weeks of treatment	Hinting task	The longitudinal arm of the study showed that TOM improved after medication was started, particularly during the first 2 weeks of antipsychotic treatment. The TOM response at 2 weeks of antipsychotic treatment reached similar values to those obtained in the cross-sectional sample
[94]	30 patients Age 20 or more Schizophrenia and schizoaffective disorder Outpatients Stable illness **	Randomized controlled pilot, open-label study	Risperidone LAI up to 50 mg/2 weeks vs Paliperidone Palmitate up to 150 mg/monthly 6 months	Baseline and after 6 months of treatment	Social Functioning Scale (SFS) Social Emotional Cognition Task (SECT)	Paliperidone may improve the total social functioning, independent life competence, and performance as compared to the Risperidone LAI group
[95]	25 patients Age 18-45 Schizophrenia Drug naïve Outpatients All stage of illness *	Short term follow-up study	Risperidone 4mg/day + trihexyphenidyl 2mg/day	Baseline and after a mean duration of 38.2±17.1 days of treatment	Tool for Recognition of Emotions in Neuropsychiatric Disorders (TRENDS)	At baseline, the patients made significantly greater errors in recognition of negative emotions of fear and disgust which improved on follow-up
[96]	20 patients Age 21-49 Schizophrenia Outpatients All stage of illness *	Longitudinal study with augmentation treatment	Perospirone range 8–32 mg/day, (mean dosage of 18.3 mg/day)	Baseline and after 6 months of treatment	Script tasks	Perospirone improved performance on the script tasks, a measure of verbal social cognition
[97]	223 patients Mean age in olanzapine group: 41.67 ± 9.53; quetiapine group: 40.45 ± 9.61 Schizophrenia and schizoaffective disorder Outpatients All stage of illness *	This study is part of a multi-site, randomized, double-blind trial	Olanzapine (mean dosage 15.6 ± 4.3 mg/day) or quetiapine (mean dosage 455.8 ± 156.3 mg/day)	Baseline and after 6 months of treatment	Social Cue Recognition Task (SCRT)	Results revealed that participants in both medicationgroups improved significantly but modestly on three out of four subscale of SCRT
[98]	20 patients Age 22-51 Mean age: 36.5 Schizophrenia or schizoaffective disorder Outpatients All stage of illness *	Randomized, double-blind, placebo-controlled, crossover trial	Adjunctive treatment of Raloxifene 120 mg/die	Baseline and after 13 weeks of treatment	Facial emotion recognition task	This study provides initial evidence suggesting that adjunctive raloxifene treatment changes neural activity in brain regions associated with facial emotion recognition in schizophrenia
[99]	60 patients Age 18-60 Mean age: 42.7 Schizophrenia Inpatients and outpatients Stable illness **	Multi-centre, double blind, placebo-controlled, randomized clinical trial	MK-0777 BID 3 or 8 mg/die	Baseline and after 4 weeks of treatment	MATRICS Consensus Cognitive Battery (MCCB)	No significant group differences on the primary outcome measure, the MCCB composite score
[100]	23 patients Age 18-56 mean age: 48.6 Schizophrenia Outpatients Stable illness **	Randomized, double-blind, placebo-controlled design	Single dose of 40 IU intranasal oxytocin (OT)	Baseline and at visit 3, 30 minutes after intranasal Oxytocine/placebo	TASIT Part III: Social Inference—EnrichedEmotional Perspective Taking Task (EPTT) Half Profile of Nonverbal Sensitivity (Half-PONS) Facial affect recognition from the standardized stimulus set developed by Ekman	OT did not significantly improve performance on the total social cognition composite measure. OT significantly improved performance in high-level social cognition (TASIT III detection of sarcasm and EPTT tests)
[101]	27 males patients Age 18-56 Mean age: 48.6 Schizophrenia Outpatients Stable illness **	Randomized, proof-of-concept, parallel group clinical trial	Single dose of 40 IU intranasal oxytocin (OT) 30 min before each social cognitive skills session	One week before receiving study drug, baseline, 1 week and 1 month after the final training on social cognitive skills	MATRICS Consensus Cognitive Battery (MCCB); The Awareness of Social Inference Test (TASIT); Half Profile of Nonverbal Sensitivity (Half-PONS); Facial affect recognition from the standardized stimulus set developed by Ekman; The empathic accuracy task; Managing emotions component of Mayer–Salovey–Caruso emotional intelligence test (MSCEIT)	OT improvement across the combined-treatment groups; significant main effects of time on individual measures of facial affect recognition, the MSCEIT Managing Emotions total score and the PONS total score. Post-treatment empathic accuracy significantly greater in OT
[102]	21 male patients Age 22-57 Mean age: 37.42 Schizophrenia or schizoaffective disorder Stable illness **	Randomized within-subjects crossover, double-blind, controlled trial	Single dose of 24 IU intranasal oxytocin (OT) spray + placebo spray 2 weeks later	Baseline 45 min after oxytocin/placebo administration and 2 weeks after treatment	Paralanguage and face subtasks of the Diagnostic Analysis of Non-Verbal Accuracy (DANVA); Facial Expressions of Emotions Task (FEEST); Reading the Mind in the Eyes Task (RMET); Facial affect recognition from the standardized stimulus set developed by Ekman; False Belief Picture Sequencing Task (FBPSTL); Hinting Task; The Faux Pas Recognition Task	OT significantly improves higher order social cognition performance above placebo. Most improvement on tests measuring appreciation of indirect hints and recognition of social faux pas
[103]	31 patients Age 18–45 Mean age: 30.4 Schizophrenia Outpatients Stable illness **	Randomized, double-blind, placebo-controlled, cross-over trial	Daily 24 IU oxytocin (OT) nasal spray	Baseline, 4 months and 8 months	Mayer– Salovey–Caruso Emotional Intelligence Test (MSCEIT); the Reading the Mind in the Eyes task (RMET); Emotional Priming Paradigm (EPP)	Significant effect of OT at MSCEIT battery, between anger and happiness recognition RTs and a significant facilitation effect
[104]	52 patients Age 16–35 Mean age: 21.92 Schizophrenia, schizophreniform disorder or schizoaffective disorder Inpatients and outpatients Less of 3 years of treatment	Randomized double-blind, placebo-controlled, between subjects trial	24 IU oxytocin nasal spray twice-daily + additional dose OT before each weekly session	Baseline, 6 weeks and 3 months	Reading the Mind in the Eyes Test (RMET); Social Functioning Scale (SFS); The Facial Expressions of Emotions Task (FEEST); tests identification of 6 basic emotions; Movie Stills Task; False Belief Picture Sequencing Task; e Faux Pas Task; Empathy Quotient; Ambiguous Intentions Hostility Questionnaire	No benefit of oxytocin nasal spray treatment on all primary and secondary outcomes

* All stages of illness: all patients independently from duration of illness and/or treatment. ** Stable illness: depending on the inclusion/exclusion criteria of each study, in general excluding acute phase of illness requiring hospitalization.

## Data Availability

MDPI Research Data Policies.
